# Therapeutic implications of epigenetic alterations in burns: a new frontier in burn medicine

**DOI:** 10.1042/CS20250585

**Published:** 2026-05-26

**Authors:** Sara Timpano, Mahmoud Farahat, Marc G. Jeschke

**Affiliations:** 1 Department of Surgery, McMaster University, Hamilton, Ontario, Canada; 2 Centre for Burn Research, Hamilton Health Sciences, Hamilton, Ontario, Canada; 3 Hamilton General Hospital, Hamilton Health Sciences, Hamilton, Canada

**Keywords:** epigenetics, immunology, inflammation, therapeutics, wound healing

## Abstract

Epigenetic modifications refer to heritable changes in gene expression that occur without alterations to the DNA sequence. The earliest evidence of DNA methylation—a key epigenetic mechanism—existed before Watson and Crick described the DNA double helix. Main forms of epigenetic regulation include DNA methylation, histone modifications, and mechanisms mediated by non-coding RNA. DNA methylation, which mainly happens at cytosine residues within cytosine-phosphate-guanine dinucleotides, is usually linked to transcriptional repression. Histone modifications, such as acetylation, methylation, and phosphorylation, affect chromatin structure and thereby alter transcriptional access. Simultaneously, non-coding RNAs like microRNAs and long non-coding RNAs regulate gene expression through transcriptional and post-transcriptional pathways. Abnormal epigenetic regulation has been strongly connected to the development of various human diseases, including cancers, diabetes mellitus, cardiovascular diseases, and neurodegenerative disorders. Recent evidence now points to epigenetic dysregulation as a key factor in the systemic and localized responses to severe burn injuries. These modifications control inflammatory signaling, immune cell polarization, and wound-healing processes. For example, DNA methylation and histone alterations in macrophages influence the balance between pro- and anti-inflammatory pathways, angiogenesis, and tissue regeneration. Additionally, severe burns are recognized as significant accelerators of epigenetic aging, with a direct link between injury severity and biological age advancement. However, the full extent of epigenetic roles in post-burn physiological changes remains poorly understood. The present review aims to explore these mechanisms and provide an insight into the complex network of factors shaping the pathophysiological response to these modifications post-burn injury.

## Introduction

**Figure 1:**
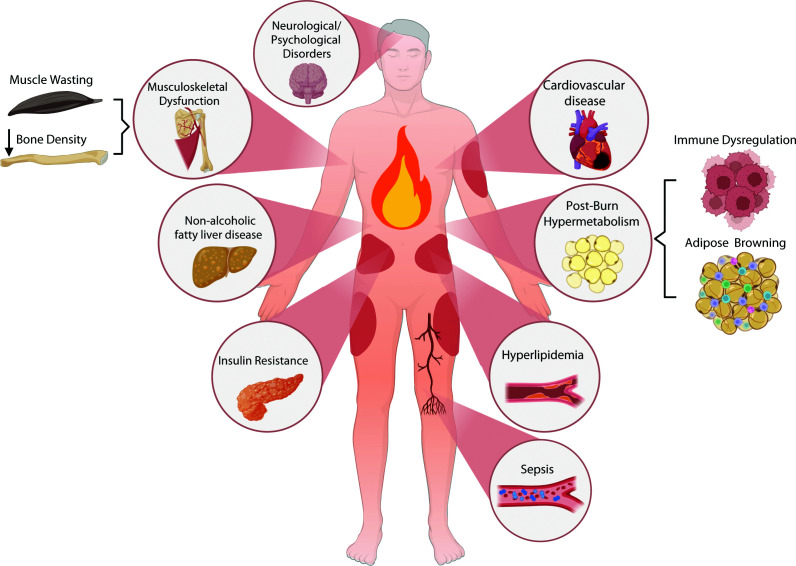
Systemic complications and metabolic consequences following severe burn injury Severe burn injury initiates a cascade of systemic responses that extend beyond the local wound site, affecting multiple organ systems. The sustained hypermetabolic and inflammatory state contributes to musculoskeletal dysfunction (including muscle wasting and loss of bone density), neurological and psychological disorders, cardiovascular disease, and immune dysregulation accompanied by adipose tissue browning. Metabolic disturbances that follow burn injury include post-burn hypermetabolism, insulin resistance, hyperlipidemia, and non-alcoholic fatty liver disease. In severe cases, this predisposes patients to sepsis and multiple organ dysfunctions. Together, these alterations reflect the widespread physiological impact of burn injury.

Burn injuries remain one of the most traumatic injuries due to the overwhelming destruction of the tissue and the associated substantial mortality and morbidity. The WHO estimates that burns account for up to 180,000 global deaths annually and 9–11 million burn injuries that require medical care [1]. Moreover, burn injuries are associated with increased risk of developing diverse comorbidities, including malignancies, cardiovascular complications, neurological disorders, metabolic diseases such as diabetes, musculoskeletal impairments, gastrointestinal pathologies, recurrent infections, and psychological conditions, including anxiety and depression [2] (Figure 1). On the other hand, a 2019 epidemiological study projected a global economic burden of $11.2 billion USD that was attributable to burns, accounting for 0.09% of the global gross domestic product [3]. Such estimates underscore burns not only as a global health burden, but a socioeconomic one as well. Severe burn injury triggers pathophysiological responses that extend far beyond the initial cutaneous damage. The ensuing cascade of immune activation, systemic inflammation, and hypermetabolism leads to severe complications such as shock, sepsis, and multiorgan failure, all considered major contributors to burn-related mortality [4]. Importantly, accumulating evidence demonstrates that the systemic response to burns is not transient but persists for years, characterized by sustained hypermetabolism and chronic inflammation [5,6]. Clinical studies have shown persistent alterations in resting energy expenditure, body composition, metabolic markers, and organ function for up to three years post-injury [5,7,8]. Elevated concentrations of stress hormones (such as cortisol and catecholamines), cytokines, and acute-phase proteins further indicate a prolonged hyperinflammatory state [5,9]. This chronic catabolic and inflammatory milieu profoundly impairs wound healing, delaying tissue repair, increasing infection risk, and predisposing to hypertrophic scarring [7]. Consequently, the biological repercussions of burn injury extend well beyond the acute phase, with ongoing disturbances in tissue repair, metabolism, and immune regulation contributing to long-term morbidity. Despite advances in critical care and reconstructive techniques, current therapies remain insufficient to correct these persistent abnormalities, underscoring the need for innovative mechanistic approaches (Figure 1). Recent findings suggest that the enduring systemic effects of severe burns may, in fact, arise from epigenetic reprogramming. Burns appear to induce stable changes in gene expression through DNA methylation (DNAm), histone modification, and non-coding RNA (ncRNA) regulation—alterations that can persist long after the initial insult. These epigenetic mechanisms have been implicated in prolonged inflammation, immune dysregulation, altered metabolism, and fibrotic remodeling [10–15]. Pharmacological modulation of these pathways using DNA methyltransferase (DNMT) and histone deacetylase (HDAC) inhibitors has been shown to reprogram fibroblast activity, modulate inflammatory signaling, and promote tissue regeneration [16–20].

Although few studies have directly characterized post-burn epigenetic alterations, parallels with other chronic inflammatory and metabolic conditions, such as diabetes and fibrotic disorders, suggest that these injuries may drive long-term phenotypic changes via persistent genetic and epigenetic remodeling. We therefore propose that the long-term physiological, metabolic, and reparative disturbances observed after severe burns are rooted in epigenetically mediated alterations of gene expression. Understanding and targeting these mechanisms may open new avenues for precision therapies aimed at restoring homeostasis, enhancing wound repair, and mitigating chronic burn sequelae.

## Overview of epigenetic mechanisms

### DNA methylation

**Figure 2:**
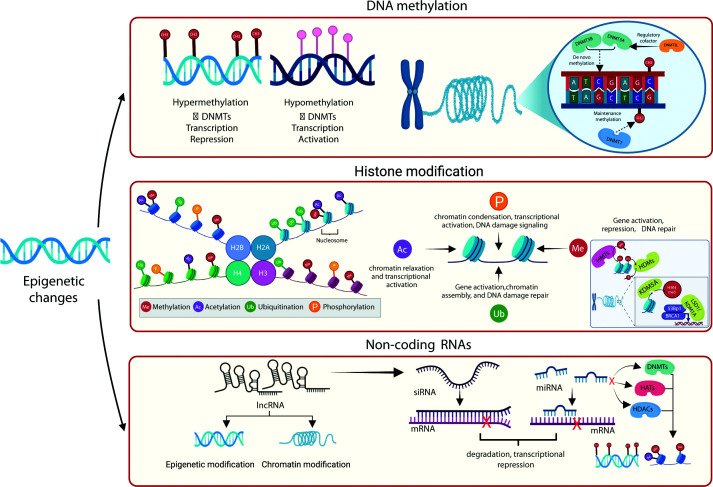
Major mechanisms of epigenetic regulation Epigenetic changes modulating gene expression without altering the underlying DNA sequence primarily involve three primary mechanisms: DNAm, histone modification, and ncRNAs. DNAm, regulated by DNMT activity, occurs predominantly at CpG sites, where hypermethylation leads to transcriptional repression, while hypomethylation is associated with transcriptional activation. Histone modifications, including methylation, acetylation, ubiquitination, and phosphorylation, alter chromatin structure and accessibility. These modifications influence nucleosome dynamics, chromatin compaction, transcriptional accessibility, and DNA repair processes. ncRNAs such as long non-coding RNAs (lncRNAs), small interfering RNAs (siRNAs), and microRNAs (miRNAs) can contribute to chromatin modifications by recruiting epigenetic enzymes (DNMTs, histone acetyltransferases (HATs), HDACs), guiding transcriptional silencing complexes, and promoting mRNA degradation or translational repression. Collectively, these mechanisms interact to maintain genomic stability and orchestrate dynamic transcriptional responses in health and disease.

DNAm constitutes a fundamental epigenetic modification that regulates gene expression, primarily through the covalent addition of methyl groups to cytosine residues within cytosine-phosphate-guanine (CpG) dinucleotides. Extensive evidence demonstrates that increased methylation within promoter and enhancer regions is generally associated with transcriptional repression, largely due to reduced accessibility of transcription factors and other regulatory proteins to their binding sites [21]. In addition to promoter elements, methylation of the first intron has also been implicated in gene expression control, underscoring the complexity of DNAm-mediated regulation across diverse genomic contexts [22]. Conversely, hypomethylation is often correlated with transcriptional activation, highlighting the dynamic interplay between methylation states and gene activity. Importantly, the reversible nature of DNAm provides a flexible mechanism for fine-tuning transcription in response to developmental signals, cellular differentiation, environmental influences, and processes related to disease susceptibility and aging [23–27] (Figure 2).

The establishment and maintenance of DNAm patterns are orchestrated by DNMTs, which play a pivotal role in shaping epigenetic landscapes critical for transcriptional regulation and genome stability [28]. Among mammalian DNMTs, DNA methyltransferase 1 (DNMT1) functions as the principal maintenance enzyme, ensuring the faithful propagation of existing methylation patterns during DNA replication and safeguarding epigenetic inheritance. By contrast, DNA methyltransferase 3A (DNMT3A) and DNA methyltransferase 3B (DNMT3B) mediate *de novo* methylation, introducing novel methylation marks particularly relevant during embryogenesis and epigenetic reprogramming [28]. Although catalytically inactive, DNA methyltransferase 3-like (DNMT3L) plays a key regulatory role by recognizing unmethylated histone H3 tails and facilitating the recruitment and activation of DNMT3A, thereby enhancing *de novo* methylation efficiency [29]. Perturbations in DNMT activity, whether due to dysregulation or mutation, can result in aberrant methylation patterns that disrupt transcriptional programs, promote genomic instability, and contribute to the development of diseases, including congenital syndromes and malignancies [28] (Table 1).

**Table 1:** Key DNA methyltransferases and their functions in the regulation of DNAm patterns

Enzyme	Main function	Role in DNAm	Key features/notes
DNMT1	Maintenance methyltransferase	Preserves existing methylation patterns during DNA replication	Ensures faithful epigenetic inheritance
DNMT3A	*De novo* methyltransferase	Establishes new methylation marks	Active during embryogenesis and epigenetic reprogramming
DNMT3B	*De novo* methyltransferase	Establishes new methylation marks	Works with DNMT3A in development and differentiation
DNMT3L	Regulatory cofactor (catalytically inactive)	Stimulates DNMT3A activity	Binds unmethylated H3 tails to promote *de novo* methylation

A summary of the major DNMTs involved in regulating DNAm, highlighting their primary functions, roles in maintaining or establishing methylation patterns, and key regulatory features. Generally, dysregulation of these enzymes leads to abnormal methylation, which causes transcriptional disruption, genomic instability, congenital disorders, and cancer.

Beyond its regulatory functions, DNAm has emerged as a robust biomarker for biological age estimation through the analysis of methylation profiles at specific CpG sites that exhibit predictable age-associated changes [30]. These molecular signatures form the foundation of epigenetic clocks, such as the widely adopted Horvath clock, which employs statistical models to predict biological age from methylation data [31]. While partially shaped by genetic inheritance, epigenetic age is highly dynamic and can be transiently accelerated by environmental exposures, disease conditions, and acute physiological stressors [32,33]. Notably, severe burn injuries have recently been identified as among the most potent accelerators of epigenetic aging [34,35], surpassing other well-established factors such as smoking, coronary artery disease, and diabetes [36–38]. These findings highlight the profound systemic effects of traumatic injury on the epigenome and underscore the potential value of DNAm-based age estimation in predicting long-term outcomes following severe burns.

### Histone modifications

Histone proteins are fundamental structural components of chromatin, providing the scaffold around which DNA is organized. DNA wraps around histone octamers—complexes composed of two copies each of histones H2A, H2B, H3, and H4—to form nucleosomes, while a fifth histone, H1, stabilizes higher-order chromatin structure by binding to linker DNA between nucleosomes [39]. The N-terminal tails of histones undergo a wide array of post-translational modifications (PTMs) that influence chromatin architecture and epigenetic regulation. These modifications (including methylation, acetylation, phosphorylation, and ubiquitination) can directly neutralize the charge of histone tails or serve as docking sites for chromatin remodeling complexes, thereby modulating DNA accessibility [40–42] (Figure 2).

Through these dynamic modifications, histones provide a versatile regulatory layer that controls gene expression without altering the underlying DNA sequence, thus serving as a central mechanism of epigenetic regulation.

#### Acetylation

HATs are a diverse group of enzymes that play a central role in regulating gene expression by catalyzing the transfer of acetyl groups from acetyl-CoA to lysine residues on histone tails. This modification neutralizes the positive charge of histones, weakening their electrostatic interaction with negatively charged DNA and thereby promoting a more relaxed chromatin structure that is permissive to transcription [40]. HATs are classified into several families based on sequence and structural homology, including the GNAT, MYST, and p300/CREB-binding protein (CBP) families, each of which contributes to distinct cellular functions [43]. By facilitating transcriptional activation, HATs are involved in essential processes such as development, cell cycle regulation, and adaptation to environmental cues [44]. Importantly, their activity is tightly regulated after recruitment to chromatin, ensuring precise control of gene expression [45]. Dysregulation of HATs has been implicated in numerous pathological conditions, underscoring their relevance as potential therapeutic targets (Table 2).

**Table 2:** Major classes of histone-modifying enzymes and their regulatory functions

Enzyme family	Classification/ families	Function	Key histone targets	Examples	Disease links	Required cofactor
Histone acetyltransferases (HATS)	GNAT family, MYST family, p300/CBP family	Add acetyl groups to lysine → neutralizes charge → chromatin relaxation → transcription activation	H3K9ac, H3K14ac, H4K16ac	GCN5 (GNAT), Tip60 (MYST), p300/CBP	Dysregulated in cancer, inflammatory diseases, neurodevelopmental disorders	Acetyl-CoA
Histone deacetylases (HDACs)	Class I, II, IV (Zn^2+^-dependent); class III (sirtuins, NAD^+^-dependent)	Remove acetyl groups → restore histone positive charge → chromatin compaction→ transcription repression	Histone H3 and H4 lysine acetylations	HDAC1, HDAC2, HDAC3, SIRT1, SIRT6	Linked to cancer, neurodegeneration, cardiovascular diseases, inflammation; SIRT1 protects against burn-induced inflammation and tissue damage	Zn^2+^ (class I/II/IV), NAD^+^ (class III)
Histone methyltransferases (HMTs)	Lysine methyltransferases (KMTS, SET domain), arginine methyltransferases (PRMTs)	Add methyl groups to lysine/arginine → does not change charge → serves as regulatory signal; outcome depends on site and methylation state	Activation: H3K4me^*^, H3K36me; Repression: H3K9me3, H3K27me3	EZH2 (KMT, H3K27), SUV39H1 (KMT, H3K9), PRMTI	Dysregulation in cancer, neurological diseases, developmental syndromes	SAM (S- adenosylmethionin e) → methyl donor
Histone demethylases	Amine oxidase family (e.g., LSD1); Jumonji C (JmjC) domain family	Remove methyl groups from lysine/arginine → dynamically regulate transcription	LSD1: H3K4me1/2; JmjC: H3K4me3, H3K9me3, H3K27me3, etc.	LSD1, KDM4A, KDM6A/B	Implicated in cancer, neurodevelopmental disorders, psychiatric conditions	FAD (LSD1), Fe^2+^/α-KG (JmjC)

These modifiers coordinate chromatin accessibility, transcriptional activation or repression, DNA repair, and epigenetic memory in physiological and pathological contexts, including burn injury and wound healing.

* Note: H3K4me3 at promoter regions is positively associated with active transcription, whereas H3K4me1 is associated with transcriptional activation when present at enhancer regions together with H3K27ac.

In contrast, HDACs counterbalance HAT activity by catalyzing the removal of acetyl groups from lysine residues on histone tails. This restores the positive charge of histones, reinforces their interaction with DNA, and promotes chromatin compaction, which limits transcriptional accessibility [46,47]. HDACs are grouped into four classes according to sequence homology and cofactor requirements: classes I, II, and IV are zinc-dependent, whereas class III, the sirtuin family, requires nicotinamide adenine dinucleotide (NAD^+^) as a cofactor [48]. Through this activity, HDACs regulate diverse processes, including gene expression, cell differentiation, and cellular responses to stress. Additionally, aberrant HDAC activity has been associated with various diseases, making them significant therapeutic targets. This highlights the broader role of HDACs beyond histone regulation, as they also modulate non-histone proteins and cellular signaling pathways that influence inflammation and tissue repair (Table 2).

#### Methylation

Histone methyltransferases (HMTs) are a family of enzymes that catalyze the transfer of methyl groups from S-adenosylmethionine to lysine or arginine residues on histone tails. They are broadly divided into two groups: lysine methyltransferases, which typically harbor a conserved SET domain, and protein arginine methyltransferases [49]. In contrast to acetylation, histone methylation does not alter the overall charge of histones; rather, it serves as a versatile signaling mechanism that influences chromatin dynamics and transcriptional outcomes [50]. The functional effect of histone methylation depends heavily on the residue modified and the extent of methylation (Figure 2). For instance, trimethylation of histone H3 at lysine 4 (H3K4me3) within promoters is a hallmark of transcriptional activation, while H3K4me1 located in enhancer regions is only associated with transcriptional activation when coupled with acetylation of histone H3 lysine 27 (H3K27ac) [51,52]. On the other hand, trimethylation at lysine 9 (H3K9me3) or lysine 27 (H3K27me3) is commonly associated with transcriptional repression and heterochromatin formation [53]. Through these modifications, HMTs play indispensable roles in development, cellular differentiation, and the maintenance of genomic integrity [54]. Dysregulation of HMT activity has been implicated in a variety of pathological conditions, including cancer, neurological diseases, and other disorders [55,56], underscoring their importance as potential therapeutic targets (Table 2).

Histone demethylases (HDMs) catalyze the removal of methyl groups from lysine or arginine residues on histone tails. These enzymes are classified into two major families: the amine oxidase family, exemplified by lysine-specific demethylase 1A (LSD1), which primarily acts on mono- and dimethylated lysines [57,58], and the Jumonji C (JmjC) domain-containing family, which is capable of demethylating all methylation states, including mono-, di-, and trimethyl marks [59]. Through this activity, HDMs play a central role in regulating transcription by dynamically influencing chromatin accessibility and the recruitment of transcriptional regulators. At this point, it is worth mentioning that members of the amine oxidase family contribute to DNA damage repair (DDR) through coordinated chromatin remodeling and recruitment of DDR proteins at damage sites, since it has been established that thermal injuries induce DNA damage to cells surrounding the wound [60]. For example, LSD1/KDM1A removes H3K4me2, to promote chromatin opening, and recruits 53BP1 and BRCA1 [61]. Similarly, KDM5A demethylates H3K4me3 to enable chromatin remodeling, while KDM4D has shown to be important for DNA break repair [62,63]. This highlights the essential role HDMs play in diverse biological processes, including embryonic development, cell fate specification, and stress responses [57]. On the other hand, aberrant regulation of HDMs has also been implicated in multiple human diseases, notably cancer and neurodevelopmental disorders; however, their direct role in burn injuries has yet to be established [55] (Table 2).

#### Phosphorylation

Histone phosphorylation plays critical roles in regulating chromatin structure and gene expression. It typically occurs on serine, threonine, or tyrosine residues within histone tails and is closely associated with cellular processes such as transcriptional activation, DNA damage response, mitotic chromosome condensation, and apoptosis [64] (Figure 2). This modification is catalyzed by various kinases, including members of the mitogen-activated protein kinase, Aurora kinase, and ataxia telangiectasia mutated kinase (ATM)/ATM and Rad3-related kinase (ATR) families, which respond to diverse extracellular and intracellular signals. Removal of these phosphate groups is mediated by phosphatases, such as protein phosphatase 1 (PP1) and protein phosphatase 2A (PP2A), restoring chromatin to its basal state once signaling cues subside [65]. Through this dynamic balance of kinase and phosphatase activity, histone phosphorylation acts as a rapid, reversible mechanism linking signal transduction pathways to chromatin-based regulation of gene expression (Figure 2).

### Non-coding RNAs

ncRNA is an umbrella term that encompasses a range of regulatory ncRNA classes that play a pivotal role in epigenetic regulation by modulating gene expression without altering the underlying DNA sequence. Among the diverse categories of ncRNAs, miRNAs, lncRNAs, and siRNAs are the best characterized in terms of their regulatory functions [66]. The miRNAs and siRNAs primarily act at the post-transcriptional level, where they directly bind to complementary mRNA sequences to promote degradation or inhibit translation, thereby down-regulating gene expression [67–70]. Interestingly, miRNA also indirectly induces epigenetic modifications by targeting key enzymes responsible for DNA and histone modifications, such as DNMTs, HDACs, and HATs at the post-transcriptional level to modulate chromatin organization, transcriptional repression, and the maintenance of cellular identity [71,72]. In contrast, lncRNAs exert their regulatory effects through multiple mechanisms, including serving as precursors for siRNAs and directing chromatin-modifying complexes, such as Polycomb repressive complexes, to specific genomic loci [66,73]. This recruitment facilitates histone modifications, alters chromatin accessibility, and can result in stable transcriptional silencing. Together, these ncRNAs form an interconnected regulatory network that fine-tunes gene expression and contributes to the maintenance of cellular function.

In addition, ncRNAs are key regulators of inflammation and wound healing, coordinating immune responses with tissue repair. miRNAs modulate inflammatory signaling by targeting cytokines and transcription factors, such as nuclear factor kappa-light-chain-enhancer of activated B cells (NF-κB) regulators, thereby controlling both initiation and resolution of inflammation [74]. The lncRNAs can influence macrophage polarization, keratinocyte migration, and fibroblast activation through interactions with chromatin modifiers or by sequestering specific miRNAs [75]. Additionally, siRNAs also hold therapeutic potential by silencing pro-inflammatory mediators and promoting wound closure [76,77].

The present review will primarily examine therapeutic strategies that modulate enzymatic regulators of epigenetic modifications, with a particular emphasis on histone modifications and DNAm. The contribution of ncRNAs to epigenetic regulation constitutes a broad and multifaceted domain that will not be explored in depth here, as it warrants comprehensive discussion in a dedicated review.

## Overview of epigenetic alterations in burn pathogenesis and burn-related conditions

Little is known about specific burn-induced epigenetic changes, and the underlying processes regulating these alterations remain largely unexplored. While accumulated reports suggest that other forms of traumatic injuries, environmental stressors, inflammation, and metabolic dysregulation can modify the epigenome [78–81], the specific molecular pathways by which burn injury affects DNAm, histone modifications, and ncRNA expression are still poorly understood. This represents a significant gap in our knowledge of how severe burn trauma influences gene regulation at the epigenetic level. Therefore, the following section will discuss not only known post-burn epigenetic modifications, but also other epigenetic alterations linked to major pathophysiological processes involved in burn injury, including persistent inflammation, excessive fibrosis, and impaired wound healing, which together contribute to chronic tissue dysfunction.

### Known epigenetic alterations following burn injury

**Figure 3:**
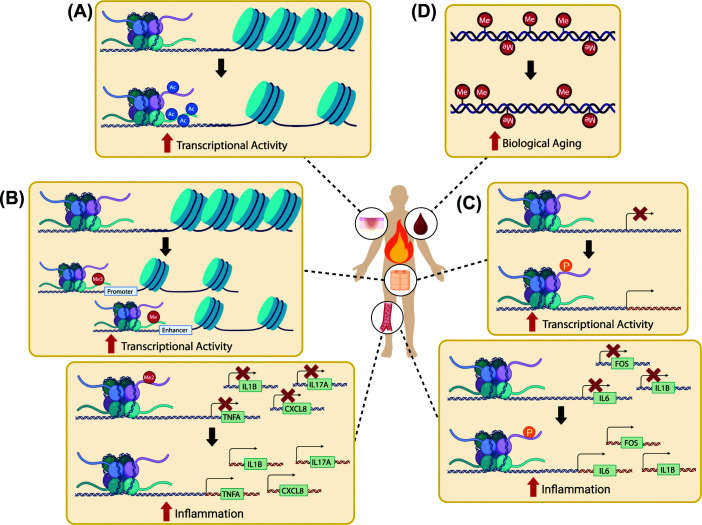
Known epigenetic alterations that directly follow burn injury Burn injury triggers epigenetic modifications that contribute to dysregulated inflammatory and metabolic responses. Of the known burn-induced modifications, these include (**A**) histone acetylation—increased levels of H3K27ac and H4K5ac, in the adjacent epidermis of the wound, along with increased H4K12ac and H4K8ac in the epithelial tongue of the wound. These histone modifications promote open chromatin and active gene transcription; (**B**) histone methylation—increased levels of mono- and tri-methylated H3K4 in the spinal dorsal horn immediately after burn are associated with open chromatin at enhancer and promoter regions, respectively, and are inferred to lead to increased transcriptional activity. Loss of the transcriptionally repressive H3K9me2 mark in promoters allows for open chromatin and leads to increased expression of inflammatory genes in circulating cells; (**C**) histone phosphorylation—increased levels of H3S10p are observed in the spinal cord immediately after burn, and increased levels of H3S28p in the promoters of inflammatory genes in circulating cells. These histone modifications promote active gene transcription; (**D**) DNA methylation—altered DNAm patterns at CpG sites linked to epigenetic clocks, in whole blood DNA, reflecting accelerated epigenetic aging. The functional consequences of these DNAm changes on chromatin organization and gene expression remain unclear.

Burn injuries have recently been shown to induce profound changes in the epigenetic landscape, including alterations in DNAm patterns and histone modifications that regulate genes critical for inflammation, cell proliferation, and tissue regeneration (Figure 3). For instance, *in vivo* studies using a porcine burn model have identified dynamic changes in histone marks such as H3K27ac, H4K5ac, H4K8ac, and H4K12ac following burn injury, all of which are associated with transcriptionally active euchromatin states [82]. Notably, these modifications exhibit distinct spatiotemporal patterns during skin healing, indicating a strong influence on key cellular and molecular repair pathways. This dynamic regulation highlights the potential of targeted modulation of these epigenetic marks to enhance tissue regeneration, modulate wound microenvironment, and ultimately accelerate wound healing and reduce recovery time as an absolute necessity in burn care.

Moreover, burn injury was shown to induce distinct epigenetic modifications in spinal neurons, characterized by the significant increase in H3K4 mono- and tri-methylation and histone H3 serine 10 phosphorylation (H3S10p), which are likely associated with the up-regulated transcriptional activation and elongation of genes implicated in nociception, the neural process of detecting and encoding noxious stimuli (i.e., thermal, mechanical, or chemical). It is important to note that although the effects of these histone PTMs on chromatin state are highly context-dependent (for example, H3S10p is associated with heterochromatin during mitosis), they are generally linked to an open chromatin state at promoter or enhancer regions [83–86]. These PTMs are selectively elevated in dynorphinergic neurons, indicating a cell-type-specific epigenetic response to burn injury, and are inferred to be involved in the activation of nociception genes [51,52,87]. Furthermore, the observed differences between the burn model and a more generalized inflammatory model suggest that burn injury triggers unique epigenetic regulatory mechanisms [87].

In addition to histone modifications, burn injury has been associated with accelerated epigenetic aging as assessed by epigenetic clocks. Interestingly, this extent of accelerated biological aging was shown to positively correlate with burn intensity and size, as demonstrated in both human and murine models [34]. Other reports confirmed that burn-induced accelerated biological aging was linked to persistent inflammation and immune dysregulation, which can further impair tissue repair [35].

These reported epigenetic shifts, although limited by the available data, provide a compelling rationale for interventions that restore a more youthful or regenerative post-burn epigenetic state, potentially improving both short-term healing outcomes and long-term tissue functionality.

### Epigenetic regulation of inflammatory responses

#### Systemic and local inflammatory responses in burns

Burn injuries elicit a robust inflammatory response, characterized by increased cytokine production [88]. While several pathways driving this response have been identified, the full scope of this process, including the detailed mechanism behind the sustained post-burn hyperinflammatory state, remains largely unknown. Understanding the epigenetic mechanisms, such as histone acetylation, that regulate cytokine expression can provide insights into the inflammatory processes following burn injuries (Figure 3).

For instance, early burn injury is associated with loss of H3 lysine 9 dimethylation (H3K9me2) in the promoters at *TNFA, IL-1B, CXCL8*, and *IL-17*, which correlates with expected changes in transcription of inflammatory genes [89]. This supports the concept that removal of repressive marks, such as H3K9me2, may de-repress inflammatory genes early after burn injury.

On the other hand, distinct histone PTM patterns were observed at promoters of inflammation-associated genes of circulating cells following severe burn injury. Specifically, there is an enrichment of histone H3 serine 28 phosphorylation (H3S28p) and H3K9me2 marks, which are linked to transcriptional activation and repression, respectively [89]. This dual modification landscape suggests a complex epigenetic regulation of inflammatory gene expression, in which dynamic interplay between activating and repressive histone marks may fine-tune the transcriptional response to burn-induced stress and systemic inflammation.

Moreover, in a mouse burn model, co-treatment with TNF-α and stearate significantly increased IL-6 gene expression and protein production in 3T3-L1 adipocytes, surpassing the effects of the individual treatments. The combined treatment led to increased acetylation of histone H3 at lysines 9 and 18 (H3K9/18ac) within the IL-6 promoter, suggesting a transcriptionally permissive chromatin state. On the other hand, inhibiting HATs with anacardic acid or curcumin treatments decreased IL-6 expression, while treatment with trichostatin A (TSA), an HDAC inhibitor, mimicked the effects of TNF-α and stearate co-treatment, enhancing IL-6 expression. The observed histone acetylation was associated with decreased levels of HDAC1 and HDAC3, indicating that TNF-α and stearate co-treatment may reduce these deacetylases, thereby promoting histone acetylation and IL-6 expression [90].

#### Epigenetic regulation of inflammatory responses in burn-related conditions

Like burn injury, chronic wounds, such as diabetic wounds, are characterized by a persistently heightened inflammatory state that impairs resolution and tissue repair. Mixed lineage leukemia 1 (MLL1), a H3K4 specific HMT, has emerged as a critical regulator of inflammatory gene transcription [91]. MLL1 promotes NF-κB-mediated activation of pro-inflammatory genes and contributes to Toll-like receptor 4 (TLR4) expression in macrophages [92,93]. In prediabetic murine models, late-stage wound macrophages displayed an increased H3K4me3 enrichment, elevated MLL1 expression, and enhanced production of inflammatory cytokines [94]. Pharmacological inhibition of MLL1 attenuates these responses, reducing inflammation and highlighting its potential as a therapeutic target in impaired wound healing [94].

During normal wound repair, interferon beta (IFN-β) signaling induces SET domain bifurcated 2 (SETDB2), an HMT, which catalyzes H3K9me3 at NF-κB target promoters. This epigenetic silence suppresses pro-inflammatory cytokines such as *IL-1β, IL-6*, and *TNF-α*, thereby facilitating resolution of inflammation and tissue repair [95]. In type 2 diabetes, however, the IFN-β-Janus kinase/STAT1 pathway is impaired, resulting in diminished SETDB2 expression and sustained NF-κB-driven inflammation Beyond cytokine regulation, SETDB2 influences purine catabolism through xanthine oxidase and uric acid pathways, and dysregulation of this axis contributes to chronic inflammation; pharmacologic targeting of SETDB2 activity or uric acid metabolism has been shown to restore healing in diabetic wound models. Moreover, aberrant activation of the STAT3/SETDB2 axis in diabetic wound macrophages alters chromatin accessibility, further perpetuating inflammatory gene expression and impairing wound resolution [94,95].

Macrophages play a pivotal role in orchestrating wound repair, mainly due to their ability to transition from a pro-inflammatory M1 to a reparative M2 phenotype. In normal wound healing, this phenotypic switch ensures resolution of inflammation and progression toward tissue remodeling. However, in chronic wounds, the M1-to-M2 transition is impaired, contributing to chronic inflammation and delayed repair [96]. In burn injury, macrophages act as master regulators of inflammation. They initially adopted a pro-inflammatory M1 phenotype, releasing cytokines such as TNF-α, IL-1β, and IL-6. As healing progresses, these cells switch toward an anti-inflammatory M2 phenotype, secreting IL-10, transforming growth factor-β (TGF-β), and growth factors such as vascular endothelial growth factor (VEGF) and platelet-derived growth factor, which promote angiogenesis, fibroblast activation, and tissue remodeling [97].

Mounting evidence has underscored the key role of epigenetic machinery in modulating macrophage polarization and, hence, their inflammatory response. For instance, KDM6B, an HDM, has been shown to influence macrophage polarization by removing repressive H3K27me3 marks at inflammatory gene promoters. Its expression is positively associated with pro-inflammatory cytokine production, yet it also facilitates chromatin remodeling that permits the transcriptional activation of M2-associated gene programs, thereby playing a dual regulatory role in the transition toward the reparative phenotype [98–100]. Similarly, the HMT, SET and MYND domain containing 3, which mediates H3K4 methylation, has been implicated in promoting M2 polarization, further underscoring the role of histone-modifying enzymes in macrophage plasticity [101].

HATs also play a central role in regulating macrophage functions. It was demonstrated that the HAT p300/CBP complex is crucial for macrophage polarization, particularly in promoting the transcription of anti-inflammatory and reparative genes associated with the M2 phenotype through histone acetylation at key enhancer regions. Similarly, it was revealed that the HAT and lysine acetyltransferase 8 (KAT8) dynamically shaped macrophage responses to environmental cues, influencing cytokine production, lipid metabolism, and inflammatory resolution [102]. Together, these findings highlight that histone acetylation status acts as an epigenetic regulator of macrophage plasticity, linking metabolic and signaling pathways to transcriptional programs that govern inflammation, tissue repair, and immune homeostasis.

Additionally, DNAm dynamics also contribute to macrophage fate determination. In diabetic wounds, epigenetic profiling has revealed a skewing of DNAm patterns that favor prolonged M1 activation. Genes associated with the pro-inflammatory M1 phenotype (*Cfb, Serping1, Tnfsf15*) are hypomethylated, supporting their sustained expression. In contrast, genes linked to the reparative M2 phenotype (*Nrp1, Cxcr4, Plxnd1, Arg1, Cdk18, Fes*) are hypermethylated, restricting their transcriptional activation and impairing the transition to a pro-healing phenotype [103].

At this point, it is worth noting that ncRNAs add another layer of regulation in macrophage polarization. For example, miR-29b, induced by TGF-β1, represses DNMT3b expression. Reduced DNMT3b activity leads to hypomethylation of the Cox-2 promoter, thereby enhancing its expression and promoting M1 polarization [104]. This miRNA-driven mechanism further contributes to the persistence of pro-inflammatory macrophages in chronic wounds. Moreover, acute burns were shown to affect numerous mRNAs and lncRNAs, inducing altered methylation patterns due to a widespread disruption of N6-methyladenosine (m6A) homeostasis in injured skin. Hypomethylated RNAs were largely down-regulated and associated with impaired wound-healing processes, while hypermethylated transcripts were up-regulated and enriched in inflammatory and cytokine signaling pathways. Key lncRNAs (e.g., *Pvt1, LINC00302, MALAT1*) and hub genes (e.g., *LOR, TGM1, CASP14*) were identified, suggesting that burn-induced m6A modifications contribute to inflammation, impaired tissue repair, and heightened infection susceptibility [105].

Finally, the transcriptional coactivator p300 plays a pivotal role in orchestrating cellular responses to hypoxia during tissue repair. It has been demonstrated that p300 is essential for the stabilization and activation of hypoxia‐inducible factor‐1α (HIF‐1α), a master regulator of genes involved in angiogenesis, metabolism, and cell survival. Under hyperglycemic conditions, impaired p300-HIF‐1α interactions diminished HIF‐1α activity and reduced expression of downstream targets necessary for effective adaptation to hypoxia. These findings underscore the importance of p300 in promoting angiogenic and reparative gene expression required for proper tissue regeneration [106]. Extending this to burn wound healing, where hypoxic stress and inflammation are pronounced, p300 may serve as a critical modulator of the regenerative response by sustaining HIF‐1α-dependent signaling. Preserving or enhancing p300 function could represent a promising strategy to improve healing outcomes.

### Role of epigenetic alterations in fibrosis

Postburn fibrosis, in the form of pathological hypertrophic scars and keloids, represents one of the most common complications following burn injuries. Although several intertwined factors contribute to excessive fibrosis and pathological scarring, the primary driver is widely attributed to the prolonged and excessive inflammation process marked by elevated levels of major proinflammatory mediators IL-6, IL-8, TNF-α, and pro-fibrotic factors such as TGF-β and connective tissue growth factor. These signals promote fibroblast-to-myofibroblast differentiation, characterized by up-regulation of α-smooth muscle actin expression and collagen deposition [107]. While these cellular and molecular mechanisms are well established, recent evidence highlighting the role of epigenetic regulation in fibrotic pathways introduces a new dimension to the understanding of post-burn fibrosis.

In human thermal-injured tissues, the lncRNA X-inactive specific transcript (XIST) has emerged as an important regulator of tissue repair through its activity as a competing endogenous RNA. Specifically, XIST acts by sponging microRNA-29b-3p (miR-29b-3p), thereby preventing it from binding to its target mRNA, collagen type I alpha 1 chain (*COL1A1*), which encodes type I collagen—a major structural protein of the extracellular matrix (ECM). Under normal conditions, miR-29b-3p suppresses *COL1A1* expression to maintain balanced ECM turnover. However, in the context of burns, XIST up-regulation diminishes the inhibitory effect of miR-29b-3p, leading to increased COL1A1 translation and enhanced collagen fiber deposition [108]. This upsurge in ECM production supports fibroblast activity, tissue remodeling, and structural stabilization of the wound bed, facilitating the restoration of dermal integrity. While this mechanism promotes wound closure and tissue repair, persistent XIST activation and excessive collagen accumulation may also predispose to fibrotic scar formation, highlighting the delicate balance between regeneration and fibrosis in the healing response.

Additionally, HDAC6, a class IIb deacetylase, supports fibrotic remodeling by enabling fibroblasts to migrate into the wound site and differentiate into collagen-producing phenotypes. HDAC6 deficiency impaired TGF-β1-induced fibroblast differentiation, reduced collagen synthesis, and led to diminished collagen deposition in skin wounds of aged mice [109]. This demonstrates that HDAC6 promotes the fibrotic phase of wound repair by facilitating fibroblast activation and ECM accumulation, and HDAC6 levels decrease with age. Taken together, these findings indicate that this mechanism may underlie the delayed or impaired wound healing, which is particularly observed in older burn patients.

### Deacetylation of non-histone proteins and burn-induced immunosuppression

In the context of burn trauma, HDAC regulation is particularly relevant; in their review, Shakespear et al*.* enumerated the pivotal roles of HDACs as key regulators of inflammation and immune function [110]. HDAC function extends beyond chromatin remodeling to influence the acetylation status of transcription factors, signaling molecules, and cytoplasmic proteins, thereby shaping immune cell differentiation, cytokine production, and inflammatory gene expression. Different HDAC isoforms exhibit context-specific functions, some promoting pro-inflammatory responses, while others facilitate immune suppression or resolution. For instance, sirtuin 1 (SIRT1), a class III HDAC, is known to increase in response to burn injury and plays a crucial role in mediating anti-inflammatory pathways [111,112].

Beyond inflammation, in the context of burn injuries, SIRT1 plays a protective role by modulating oxidative stress and cellular survival pathways [113–117]. Following severe burns, SIRT1 activity is frequently diminished, a reduction that contributes to heightened inflammatory signaling and exacerbated tissue damage. Experimental studies have demonstrated that restoring or enhancing SIRT1 activity attenuates inflammation, supports tissue repair, and improves overall outcomes [118–121]. Mechanistically, these effects are mediated through the deacetylation of key transcription factors such as NF-κB and p53, which regulate inflammatory and apoptotic responses, respectively [119]. Given its multifaceted role in promoting cellular resilience and repair, SIRT1 has emerged as a promising therapeutic target for mitigating complications and improving recovery after burn trauma.

In addition to its role in fibrosis, HDAC6 regulates both histone and non-histone substrates and plays multiple roles in immune cell function, inflammatory signaling, and protein quality control. Key mechanisms include deacetylation of α-tubulin (impacting microtubule stability and cell motility), regulation of heat shock protein 90 and its client proteins, modulation of Foxp3 in T regulatory (Treg) cells, and trafficking/activation of inflammasomes (notably NOD-, LRR- and pyrin domain-containing protein 3) via its ubiquitin‐binding capacity [122]. This emphasizes the therapeutic potential of HDAC inhibitors and/or activators through modulating aberrant immune activation, chronic inflammation, and wound healing.

## Potential therapeutic targets of epigenetic modifications in burns

In pathological conditions that share features with burn injury, such as chronic wounds, diabetic ulcers, and fibrotic disorders, therapeutic modulation of epigenetic regulators has shown promising results in promoting tissue regeneration, reducing excessive inflammation, and enhancing ECM remodeling (Table 3). Although these approaches have not yet been tested clinically in burn patients, the mechanistic similarities in tissue damage, hypoxia, and inflammatory dysregulation suggest significant therapeutic potential. Targeting epigenetic pathways offers a strategy to fine-tune cellular responses in the wound microenvironment, potentially accelerating healing, preventing chronicity, and limiting pathological scarring.

**Table 3:** Epigenetic enzyme modulation and therapeutic implications in burn and wound healing

Target/enzyme	Compound/drug	Mechanism of action	Model	Experimental result	Therapeutic implications	Reference
HATs (e.g., PCAF, KAT8)	SPV-106	Activates HAT PCAF → ↑ histone lysine acetylation	Preclinical murine model (mouse wound model)	Enhances acetylation and accelerates wound repair via an NO-independent mechanism	Promotes tissue regeneration through targeted HAT activation	[123]
	MG149	Inhibits KAT8 (acetyltransferase) → ↓ H4K16ac	Preclinical murine model (mouse allergic asthma model)	Decreased pro-inflammatory gene expression, ↓collagen deposition, and ↓ inflammatory cell infiltration	Highlights KAT8-dependent acetylation in inflammation and repair	[124]
HDACs (HDAC1-10)	TSA (trichostatin A)	Broad-spectrum HDAC inhibitor (targets HDAC1, 3, 4, 6, 10)	Preclinical murine model (mouse cecal ligation and puncture (CLP) model)	Induces M2 macrophage polarisation; ↓ TNF-α, ↓ IL-6, ↓ IL-1β expression	Attenuates inflammation and promotes reparative macrophage function	[126,127]
	Tubastatin A	Selective HDAC6 inhibitor	Preclinical murine model (diabetic mouse model)	Accelerates wound healing; ↓ neutrophil/T cell infiltration, ↓ IL-1β; ↑ collagen deposition and angiogenesis	Reduces inflammation and enhances tissue repair	[128]
	Vorinostat (SAHA)	Broad-spectrum HDAC inhibitor (targets HDAC1, 2, 3, 6)	Clinical human (kidney transplant patients)	↓ pro-inflammatory cytokines (↓ TNF-α, ↓ IL-6, ↓ IL-1β); ↑ Treg activity and Foxp3 demethylation	Approved cancer drug with potential for wound-healing enhancement	[129]
	Valproic acid (VPA)	Inhibits class I/II HDACs → ↑↑ H3/H4 acetylation	Preclinical murine model (mouse wound model)	Promotes wound closure; ↓ TNF-α, ↓ IL-6, and ↓ IL-1β; ↑ IL-10, ↑ TGF-β1, ↑ VEGF; enhances fibroblast/endothelial migration	Broad immunomodulatory and pro-regenerative effects	[131]
HMTs (e.g., MLL1)	MI-2	Inhibits MLL1 (H3K4 methyltransferase)	Preclinical murine model (diabetic mouse model)	↓ CD4^+^ T cell activation; ↓ inflammation in diabetic wounds	Restores balanced immune response and improves repair	[133]
HDMs (e.g., KDM6A/B)		Removes repressive H3K27me3 marks	Preclinical murine model (mouse wound model)	Up-regulation at wound edges promotes Myc and Egfr activation; facilitates repair	Activation supports gene expression for regeneration	[134]
DNMTs (e.g., DNMT1)	siWTAP / DNMT1 knockdown	Reduces DNMT1 expression and methylation activity	Preclinical murine model (diabetic mouse model)	Accelerates wound closure, improves re-epithelialization, and collagen deposition	Reprogramming of the epigenetic landscape toward regeneration	[135,136]
	5-azacytidine (5-Aza)	Nucleoside analog; inhibits DNMT1 → DNA hypomethylation	Preclinical murine model (rat wound model)	Enhances epithelial differentiation; ↑ TGF-β, ↑ involucrin, cytokeratin; ↓ TNF-α, IL-10	Promotes pro-regenerative gene expression and tissue repair	[138,139]

Key epigenetic targets and their pharmacological modulators that influence inflammation, macrophage polarization, and tissue repair.

### Targeting HAT and HDAC activity

In the context of burn injuries, dysregulated histone acetylation can exacerbate inflammatory responses and impair wound healing. Pharmacological modulation of HATs and HDACs has emerged as a promising strategy to restore balanced gene expression, attenuate excessive inflammation, and promote reparative processes in damaged tissues. By selectively targeting these enzymes, it may enable precise macrophage polarization, cytokine production, and ECM remodeling, offering a novel avenue for improving burn wound outcomes.

Direct activation of the HAT p300/CBP-associated factor (PCAF) using the small-molecule activator pentadecylidenemalonate 1b (SPV-106) has been shown to increase lysine acetylation within the wound tissue. Remarkably, this epigenetic modulation was sufficient to accelerate the repair process through a nitric oxide-independent mechanism, highlighting the potential of targeted HAT activity enhancement as a potential therapeutic strategy for enhancing burn wound healing [123].

**Figure 4:**
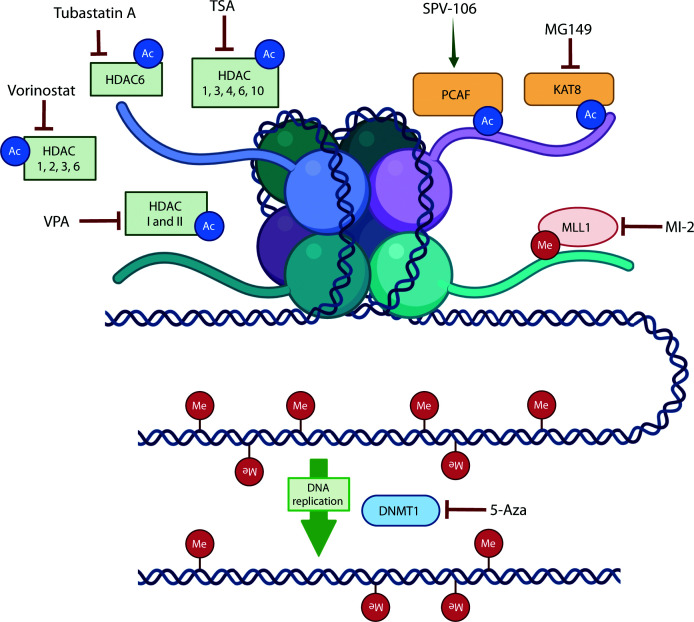
Pharmacological modulation of epigenetic enzymes These agents illustrate therapeutic strategies to manipulate epigenetic marks and regulate gene expression.

Moreover, pharmacological inhibition of KAT8 using the selective acetyltransferase inhibitor MG149 revealed that KAT8 activity is essential for maintaining appropriate H4 lysine 16 acetylation (H4K16ac) and the transcription of genes involved in oxidative metabolism and immune regulation. Loss of KAT8 activity disrupted mitochondrial function, reduced oxidative phosphorylation, and skewed macrophages toward a more pro-inflammatory phenotype [124]. This suggests that modulation of KAT8-dependent acetylation may represent a promising avenue for regulating inflammation and tissue repair in pathological contexts such as burn wound healing [125] (Figure 4).

Several HDACs, including HDAC1, 2, 3, 6, and 8, are involved in regulating macrophage polarization. The broad-spectrum HDAC inhibitor TSA, which targets HDAC1, 3, 4, 6, and 10, has been shown to promote polarization toward the M2 phenotype [126]. TSA treatment reduces the expression of pro-inflammatory cytokines such as TNF-α, IL-6, and IL-1β in macrophages as well as surface markers associated with mature, activated macrophages, thereby modulating both functional and phenotypic aspects of macrophage activation [127].

In diabetic mouse models, treatment with the selective HDAC6 inhibitor Tubastatin A accelerated wound healing. This intervention reduced the infiltration of neutrophils, T cells, and macrophages, while simultaneously promoting collagen deposition, angiogenesis, and the expression of fibrotic factors. Additionally, Tubastatin A-treated wounds exhibited decreased levels of the pro-inflammatory cytokine IL-1β, highlighting its dual role in dampening inflammation and enhancing tissue repair [128].

Vorinostat, a broad-spectrum HDAC inhibitor targeting HDAC1, 2, 3, and 6, has demonstrated potent anti-inflammatory effects in clinical settings. In kidney transplant patients, vorinostat treatment significantly lowered the levels of pro-inflammatory cytokines IL-1β, TNF-α, and IL-6 in peripheral blood mononuclear cells (PBMCs) for up to 100 days post-transplant. This treatment also increased Treg numbers, induced demethylation of the *Foxp3* promoter, and boosted Treg suppressive activity. These Tregs exhibited elevated expressions of CD45RA and CD31, indicative of a more functionally active, suppressive phenotype [129]. While vorinostat is currently approved for cancer therapy, its immunomodulatory properties suggest potential applications in other inflammatory conditions. Notably, preclinical studies indicate that vorinostat may also significantly accelerate wound healing when combined with paclitaxel, a mitotic inhibitor, highlighting its promise as an adjunctive therapy to promote tissue repair [130] (Figure 4).

Valproic acid (VPA), which inhibits class I and II HDACs and increases acetylation of histones H3 and H4, has demonstrated potent effects on wound healing by modulating inflammatory and reparative processes. In mouse models, VPA-treated PBMCs enhanced wound closure, reduced levels of pro-inflammatory cytokines TNF-α, IL-6, and IL-1β, and boosted anti-inflammatory mediators IL-10 and TGF-β1. VPA treatment also decreased the number of apoptotic cells in the wound and enhanced macrophage phagocytic activity [131]. Furthermore, exosomes derived from VPA-treated cells promoted the proliferation and migration of human skin fibroblasts and human umbilical vein endothelial cells, suppressed the expression of MMP-9, IL-1β, IL-8, TNF-α, and prostaglandin E2, and increased VEGF expression. *In vivo*, VPA-EXO reduced inflammation at the wound site, accelerated wound healing, and significantly increased collagen deposition, highlighting its multifaceted role in promoting tissue repair [132] (Figure 4).

### HMT inhibition and HDM dysregulation in inflammation and impaired wound healing

Dysregulation of HMTs and HDMs has been implicated in chronic inflammation, impaired macrophage polarization, and defective tissue repair, all of which contribute to poor wound-healing outcomes. Pharmacological inhibitors targeting these enzymes can rebalance inflammatory and reparative gene programs, restore macrophage plasticity, and promote resolution of chronic wounds.

MLL1 deposits H3K4me3 marks on promoters of cytokines and other immune mediators in macrophages to coordinate the inflammatory response. In the context of diabetes and prediabetes, MLL1 expression is dysregulated—initially suppressed and subsequently pathologically elevated—leading to erratic and prolonged inflammation within wound sites [94]. Beyond macrophage regulation, MLL1 directly modulates cluster of differentiation 4-positive (CD4^+^) T cell differentiation by controlling expression of the Th17 transcription factor retinoic acid receptor-related orphan receptor gamma through an H3K4me3-dependent mechanism, while also enhancing Notch receptor signaling, which is required for proper CD4^+^ T cell development and wound repair. This dual role of MLL1 in both innate and adaptive immune responses contributes to sustained inflammation in diabetic wounds. Importantly, pharmacological inhibition of MLL1 with the small-molecule inhibitor MI-2 has been shown to reduce CD4^+^ T cell numbers and attenuate inflammation, highlighting its therapeutic potential in restoring balance to the wound-healing process [133].

HDM inhibition is generally associated with impaired or delayed tissue repair. During cutaneous wound repair, the H3K27 demethylases, KDM6B and KDM6A, are up-regulated at the wound edge. This induction coincides with the down-regulation of Polycomb group proteins such as embryonic ectoderm development, enhancer of zeste homolog 2 (Ezh2), and suppressor of zeste 12, resulting in a reduction of the repressive H3K27me3 in mouse kin models [134]. The loss of this repressive mark facilitates the transcriptional activation of genes central to repair processes, including Myc and epidermal growth factor receptor (Egfr). These findings highlight a dynamic epigenetic shift from transcriptional repression toward activation at key regulatory regions, likely involving enhancer elements.

### DNA methyltransferase inhibitors

DNMT inhibition (DNMTi) represents a promising class of epigenetic modulators with potential applications in wound repair, including burn injury. By blocking DNMTs, these agents reduce aberrant DNAm and promote the reactivation of silenced genes involved in regeneration, inflammation resolution, and tissue remodeling. Although primarily studied in oncology, emerging evidence suggests that DNMTi may enhance repair capacity in damaged tissues by reprogramming the epigenetic landscape toward a more permissive transcriptional state. The therapeutic relevance of burn wounds remains an emerging field of investigation, offering opportunities to modulate scar formation and improve functional outcomes.

Aberrant activation of DNMT1 has emerged as a critical barrier to effective tissue repair, particularly in diabetic wounds. Hyperglycemia and oxidative stress up-regulate pathways such as the Wilms tumor 1-associated protein (WTAP)–DNMT1 axis, where increased methylation of *DNMT1* mRNA enhances its stability and expression in endothelial cells [135]. Elevated DNMT1 activity leads to hypermethylation and silencing of genes essential for angiogenesis, epithelial migration, and ECM remodeling, thereby contributing to endothelial dysfunction and impaired repair. Experimental knockdown of WTAP through siRNA, which lowers DNMT1 expression, has been shown to accelerate wound closure, promote re-epithelialization, and improve collagen deposition in diabetic models [135,136]. These findings highlight DNMT1 inhibition—either directly or via upstream regulators—as a promising strategy to restore a pro-regenerative transcriptional landscape and enhance wound-healing outcomes (Figure 4).

DNMT1 overexpression in hematopoietic stem cells (HSCs) has also been implicated in defective immune responses during diabetic wound healing. In type 2 diabetes mouse models, oxidative stress elevates DNMT1 levels in HSCs, driving hypermethylation of transcription factors such as *Notch1, PU.1*, and *Krüppel-like factor 4* that are critical for macrophage lineage commitment. This epigenetic repression reduces macrophage recruitment to the wound site and skews polarization toward a persistent pro-inflammatory M1 phenotype, ultimately delaying repair. Notably, experimental knockdown of DNMT1 in HSCs from diabetic mice restored macrophage numbers, rebalanced polarization, and significantly improved healing outcomes, underscoring DNMT1 inhibition as a strategy to correct immune dysfunction in chronic wounds [137].

5-azacytidine (5-Aza; also known as azacitidine), a nucleoside analog that also inhibits DNMT1, has shown therapeutic potential in enhancing wound repair [138]. In a rat wound model, topical 5-Aza application once daily significantly accelerated wound closure. Histological analyses demonstrated efficient re-epithelialization and enhanced cellular proliferation, as confirmed by BrdU incorporation. At the molecular level, treatment was associated with increased expression of TGF-β, involucrin, and cytokeratin, alongside reduced levels of the pro-inflammatory cytokines TNF-α and IL-10 [139]. Collectively, these findings suggest that DNMTi via 5-Aza promotes a pro-regenerative environment by stimulating epithelial differentiation and dampening excessive inflammatory signaling (Figure 4).

### Challenges in epigenetic therapy

Despite the growing promise of epigenetic modulators in wound healing and regenerative medicine, several challenges limit their clinical translation. One of the foremost concerns is specificity. Epigenetic enzymes, such as DNMTs and histone modifiers, regulate broad networks of genes across multiple tissues. Systemic inhibition of these enzymes' risks affecting non-target pathways, potentially leading to unwanted immune modulation, oncogenic activation, or disruption of normal tissue homeostasis. Designing compounds that selectively modulate target loci without perturbing global chromatin architecture remains a key hurdle.

Another critical barrier is drug delivery and off-target effects. Most epigenetic drugs currently used in oncology are delivered systemically, raising concerns about bioavailability, toxicity, and unintended tissue exposure. In the context of wound healing, local delivery strategies such as topical formulations, hydrogels, or nanoparticle-based systems offer a potential solution; however, achieving controlled release, stability, and penetration into the wound microenvironment is a complex task. Off-target effects may not only compromise safety but could also exacerbate scarring or fibrosis, undermining therapeutic benefit.

A further challenge lies in inter-individual variability in epigenetic landscapes. Epigenetic marks are influenced by genetic background, age, metabolic status, and environmental exposures, leading to significant patient-to-patient heterogeneity. For example, diabetic wounds are characterized by distinct DNAm and histone PTM patterns compared with acute traumatic wounds. This variability complicates the development of universal therapeutic strategies and highlights the need for precision approaches that integrate epigenomic profiling. Personalized epigenetic therapies may ultimately be required, but this raises practical challenges related to biomarker discovery, diagnostic testing, and regulatory approval.

These challenges underscore the complexity of translating epigenetic therapies from bench to bedside. Overcoming them will require the development of highly specific modulators, innovative delivery systems tailored to wound microenvironments, and strategies that account for patient-specific epigenetic variability.

## Current research and clinical outlook

Although clinical applications of epigenetic therapies in burn care are still in the early stages, several strategies are currently under investigation. These include small molecule modulators targeting DNMTs, HATs, HDACs, HMTs, and HDMs. Combining these epigenetic modulators with conventional burn wound therapies may provide synergistic benefits by optimizing the wound microenvironment and enhancing regenerative responses.

Circulating epigenetic biomarkers, including cell-free DNA (cfDNA) and miRNAs, offer a non-invasive approach to monitor wound healing and predict clinical outcomes. Specific miRNAs have been identified as key modulators of burn repair; for instance, miR-21 promotes dendritic cell differentiation by inhibition of phosphatase and tensin homolog and stimulation of the AKT/phosphoinositide 3-kinase pathway, while miR-129-2-3p regulates immune responses in diabetic wound models [140]. CfDNA carries methylation signatures that reflect the epigenetic state of multiple tissues and can be used to estimate biological age. In burn patients, cfDNA-based epigenetic clocks provide a dynamic measure of systemic stress and regenerative capacity, offering prognostic insight into the risk of delayed healing or chronic complications [34]. Integrating these biomarkers into clinical practice could enable patient stratification, early identification of high-risk individuals, and real-time adjustment of therapeutic strategies.

Burn injuries exhibit wide inter-individual variability in severity, immune response, and healing capacity, highlighting the importance of personalized medicine approaches. Genomic and epigenomic profiling—including DNAm patterns and histone PTMs—can reveal patient-specific differences in inflammatory response, angiogenesis, and tissue regeneration. Combining molecular insights with clinical parameters allows stratification into risk categories for delayed healing, excessive scarring, or infection. This personalized framework can guide the selection and timing of targeted interventions, including epigenetic modulators, growth factors, or cell-based therapies, ultimately improving wound closure, minimizing complications, and enhancing both functional and aesthetic outcomes.

## Future directions and perspectives

**Figure 5:**
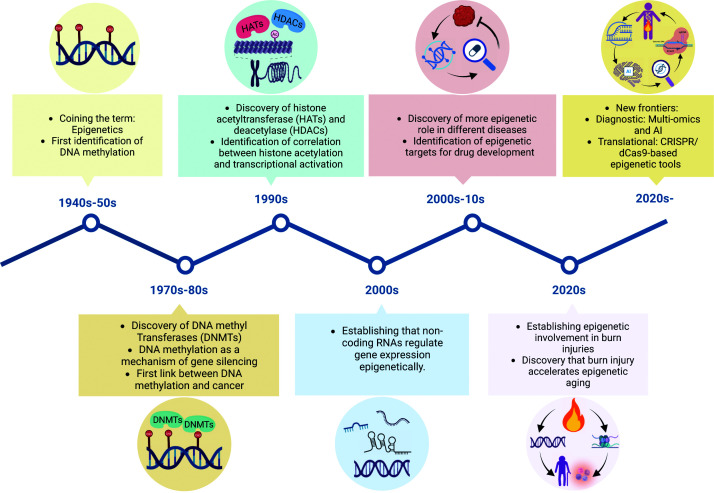
Milestones in the epigenetic field and future translational opportunities This timeline illustrates key milestones in the field of epigenetics, from the initial identification of DNAm in the 1940s to the emergence of advanced epigenetic editing tools in the 2020s. Major discoveries include the characterization of DNMTs, HATs, and HDACs, as well as the recognition of ncRNAs as epigenetic regulators. Recent insights linking epigenetic dysregulation to burn injury and accelerated biological aging highlight the growing translational potential of epigenetic research for diagnostics and therapeutics in tissue repair and regeneration.

The rapid and significant progress in the epigenetics field over recent decades offers an optimistic outlook for future burn care. Burn wound healing is poised for rapid advancement driven by the integration of multi-omic approaches. Combining epigenomic profiling with transcriptomic and proteomic analyses allows for a comprehensive understanding of the regulatory networks that govern inflammation, angiogenesis, and tissue regeneration. Such integrative strategies can identify critical epigenetic nodes that directly influence gene and protein expression, enabling more precise targeting of therapeutic interventions (Figure 5).

Emerging genome-editing technologies, particularly clustered regularly interspaced short palindromic repeats/deactivated Cas9-based epigenetic tools, offer unprecedented opportunities for locus-specific modulation of chromatin states. By recruiting activators or repressors to defined genomic regions without altering the underlying DNA sequence, these approaches can selectively up-regulate regenerative genes or silence pro-inflammatory pathways, potentially minimizing off-target effects observed with global epigenetic modulators. Their application in burn wounds could allow for highly targeted enhancement of re-epithelialization, angiogenesis, or collagen deposition.

Despite promising preclinical data, a significant gap exists in the form of long-term human studies. Most current evidence comes from animal models or *in vitro* experiments, which limit our understanding of individual differences, long-term outcomes, and potential systemic effects of epigenetic therapies in burn patients. Prospective, long-term studies are crucial for validating biomarkers, evaluating therapeutic effectiveness, and determining optimal dosing and delivery methods.

Finally, the translation of epigenetic therapeutics into clinical practice must address regulatory and ethical considerations. Locus-specific editing, systemic epigenetic modulation, and personalized approaches raise questions regarding safety, long-term consequences, and equitable access. Robust frameworks for clinical trials, monitoring, and ethical oversight will be critical to ensure that these innovative therapies are both effective and responsibly implemented.

Collectively, the convergence of multi-omic profiling, precision epigenetic editing, and well-designed human studies offers a roadmap for the development of next-generation therapeutics that can transform burn care and optimize regenerative outcomes.

## Open Access

Open access for this article was enabled by the participation of McMaster University in an all-inclusive Read & Publish agreement with Portland Press and the Biochemical Society under an agreement with OCUL.

## Abbreviations

5-Aza: 5-azacytidine (azacitidine)
AKT: protein kinase B
ATM: ataxia telangiectasia mutated kinase
ATR: ATM and Rad3-related kinase
CBP: CREB-binding protein
CD4^+^: cluster of differentiation 4-positive
cfDNA: cell-free DNA
COL1A1: collagen type I alpha 1 chain
CpG: cytosine-phosphate-guanine
DDR: DNA damage repair
DNAm: DNA methylation
DNMT: DNA methyltransferase
DNMT1: DNA methyltransferase 1
DNMT3A/3B: DNA methyltransferase 3A/3B
DNMT3L: DNA methyltransferase 3-like
ECM: extracellular matrix
Egfr: epidermal growth factor receptor
Ezh2: enhancer of zeste homolog 2
H3K27ac: acetylation of histone H3 lysine 27
H3K27me3: trimethylation of histone H3 lysine 27
H3K4me1/2/3: mono-, di-, or trimethylation of histone H3 lysine 4
H3K9me2/3: di- or trimethylation of histone H3 lysine 9
H3S10p/H3S28p: phosphorylation of histone H3 at serine 10/28
H4K5ac/H4K8ac/H4K12ac/H4K16ac: acetylation of histone H4 lysine 5/8/12/16
HAT: histone acetyltransferase
HDAC: histone deacetylase
HDM: histone demethylase
HIF-1α: hypoxia-inducible factor 1-alpha
HMT: histone methyltransferase
HSCs: hematopoietic stem cells
IFN-β: interferon beta
IL: interleukin
JmjC: Jumonji C
KAT8: lysine acetyltransferase 8
KDM: lysine demethylase
lncRNA: long non-coding RNA
LOR: loricrin
LSD1 (KDM1A): lysine-specific demethylase 1A
m6A: N6-methyladenosine
MALAT1: metastasis-associated lung adenocarcinoma transcript 1
MG149: selective KAT8 inhibitor
MI-2: mixed lineage leukemia 1 inhibitor
miRNA: microRNA
MLL1: mixed lineage leukemia 1
MMP-9: matrix metalloproteinase 9
Myc: MYC proto-oncogene
NAD^+^: nicotinamide adenine dinucleotide
ncRNA: non-coding RNA
NF-κB: nuclear factor kappa-light-chain-enhancer of activated B cells
Notch1: Notch receptor 1
PBMC: peripheral blood mononuclear cell
PCAF: p300/CBP-associated factor
PDGF: platelet-derived growth factor
PGE2: prostaglandin E2
PI3K: phosphoinositide 3-kinase
Plxnd1: Plexin D1
PP1: protein phosphatase 1
PP2A: protein phosphatase 2A
PTM: post-translational modification
Pvt1: plasmacytoma variant translocation 1
RORγ: retinoic acid receptor-related orphan receptor gamma
SETDB2: SET domain bifurcated 2
siRNA: small interfering RNA
SIRT1: sirtuin 1
SPV-106: pentadecylidenemalonate 1b
STAT: signal transducer and activator of transcription
TGF-β: transforming growth factor beta
Th17: T helper 17 cells
TLR4: Toll-like receptor 4
TNF-α: tumor necrosis factor alpha
Treg: T regulatory
TSA: trichostatin A
VEGF: vascular endothelial growth factor
VPA: valproic acid
WTAP: Wilms tumor 1-associated protein
XIST: X-inactive specific transcript
